# A representation-level wavelet–EMD fusion framework for spectral analysis and detection of epileptiform patterns in pediatric EEG: computational validation with a roadmap for external benchmarking

**DOI:** 10.3389/fnins.2026.1884876

**Published:** 2026-10-14

**Authors:** Dan Feng, Zhengtao Li

**Affiliations:** Department of Pediatric Neurology and Respiratory Medicine, Central Hospital of Enshi Tujia and Miao Autonomous Prefecture, Enshi City, Hubei, China

**Keywords:** electroencephalography, empirical mode decomposition, machine learning, pediatric epilepsy, representation-level fusion, seizure detection, time–frequency analysis, wavelet transform

## Abstract

**Background:**

Pediatric EEG analysis for epilepsy detection is challenged by the non-stationary and developmentally heterogeneous nature of epileptiform activity. Conventional Fourier approaches provide limited time–frequency localization, whereas single-domain methods such as the wavelet transform and empirical mode decomposition (EMD) each exhibit complementary weaknesses.

**Methods:**

We developed a representation-level wavelet–EMD fusion framework that integrates wavelet and EMD spectra before feature extraction. The pipeline included adaptive wavelet denoising, energy-criterion EMD, weighted time–frequency fusion, extraction of eight spectral–temporal features, and classification using a soft-voting ensemble of a radial basis function support vector machine, random forest, and neural network. Evaluation was performed on 2,000 synthetic pediatric EEG segments spanning canonical epileptiform morphologies and multiple signal-to-noise ratios. A feature-level fusion variant was implemented as an ablation comparator to isolate the effect of fusion strategy while preserving identical feature extraction, classifier architecture, and validation procedures.

**Results:**

Across 10-fold stratified cross-validation with 100 Monte Carlo replications, the proposed framework achieved 94.6 ± 0.6% accuracy, 93.7 ± 0.8% sensitivity, 95.5 ± 0.5% specificity, F1 score of 0.943 ± 0.007, and AUROC of 0.971 (95% CI, 0.962–0.978). Performance exceeded standalone wavelet, EMD, and Fourier baselines (all *p* < 0.001). Representation-level fusion also outperformed feature-level fusion by 1.4 percentage points in accuracy, with the greatest gains observed for polyspike and slow-wave morphologies. On the synthetic corpus the framework was competitive with EEGNet and a 1D convolutional network while retaining explicit time–frequency interpretability. In an external feasibility validation on the CHB-MIT pediatric scalp EEG corpus (14 subjects, leave-one-subject-out), representation-level fusion attained the highest performance of all methods (AUROC 0.901, accuracy 86.7%), confirming that the fusion strategy transfers to real recordings, with performance decreasing as expected.

**Conclusion:**

Representation-level wavelet–EMD fusion provides an interpretable and transparent computational framework for analyzing non-stationary pediatric epileptiform EEG whose advantage over classical time–frequency baselines persists, at feasibility scale, on real CHB-MIT recordings and remains competitive with deep-learning baselines. Because the primary evaluation is synthetic and the external validation is a feasibility-scale demonstration, these figures should be read as evidence of methodological viability and external transportability rather than definitive clinical performance; validation on larger, multi-center and prospective pediatric cohorts is the essential next step.

## 1 Introduction

Pediatric epilepsy represents one of the most prevalent neurological disorders of childhood, affecting between 0.5 and 1% of children worldwide (Aaberg et al., 2017). Timely and accurate diagnosis is essential because uncontrolled seizures during critical developmental windows can produce lasting deficits in cognition and behavior (Srivastava et al., 2021). Electroencephalography (EEG) continues to serve as the principal diagnostic and monitoring tool for pediatric epilepsy, offering noninvasive access to cortical electrical activity at high temporal resolution (Shellhaas, 2015). However, the interpretation of pediatric recordings is complicated by ongoing cortical maturation, frequent movement and muscle artifacts, and substantial morphological heterogeneity across epilepsy syndromes and age groups (Whelehan et al., 2018).

Visual inspection by trained electroencephalographers remains the reference standard but is labor-intensive and exhibits notable interobserver variability for subtle interictal discharges (Britton et al., 2016). These limitations have motivated extensive research into computational methods that supplement clinical reading. Conventional Fourier-based spectral analysis offers limited utility because it assumes signal stationarity, an assumption violated by epileptiform events whose frequency content evolves rapidly. Modern automated systems therefore depend on time–frequency representations that adapt to local signal characteristics.

Among time–frequency techniques, the wavelet transform has become a dominant analytic framework for non-stationary biomedical signals (Faust et al., 2015). Through multiresolution decomposition, the wavelet transform represents signals across nested scales, permitting simultaneous analysis of slow rhythms and rapid transients (Adeli et al., 2003; Subasi, 2007). The Daubechies family, and in particular the db4 wavelet, has demonstrated empirically favorable performance for spike and sharp-wave detection because its asymmetric profile approximates the morphology of epileptiform transients (Kumar et al., 2017). Nevertheless, wavelet performance depends critically on the chosen mother wavelet, and a fixed basis cannot fully accommodate the morphological diversity of pediatric epileptic patterns. Empirical mode decomposition (EMD), introduced by Huang et al. (1998), provides a complementary, fully data-driven alternative that adaptively partitions a signal into intrinsic mode functions without prior assumptions regarding basis composition and has been applied successfully to seizure detection (Riaz et al., 2016). Its adaptive nature captures slow amplitude modulations that wavelet bases may fail to isolate cleanly, although it is sensitive to boundary effects and to mode mixing, particularly when high-frequency transients are superimposed on slowly varying backgrounds.

Recognizing the complementary strengths of these decompositions, several investigators have explored hybrid pipelines. Jana et al. (2022) reported a feature-level fusion of the discrete wavelet transform and EMD that improved seizure detection on the Bonn dataset, and Du et al. (2024) combined complete ensemble EMD with the continuous wavelet transform for denoising of epileptic EEG. Although these hybrid approaches improve over single-method baselines, they share an architectural choice that has not been systematically scrutinized: fusion occurs after independent feature extraction from each transform. Cross-domain information that lives in the joint spectrum is therefore discarded before the classifier sees it. In parallel, deep learning models have become a predominant approach in adult seizure detection (Acharya et al., 2018; Nafea and Ismail, 2022), but their translation to pediatric EEG has lagged because of limited annotated data and strong inter-subject variability.

In the present study, we develop and computationally validate a representation-level wavelet–EMD fusion framework that performs integration directly in the time–frequency domain. The framework is benchmarked against three single-domain baselines (Fourier–Welch, wavelet alone, EMD alone) and against a feature-level fusion ablation that isolates the contribution of representation-level fusion. We frame the work as a methodological contribution that establishes a transparent, fully reproducible computational baseline and isolates, through a controlled ablation, the effect of performing wavelet–EMD fusion at the representation level rather than the feature level. We benchmark the framework against classical time–frequency baselines and, as a secondary comparison, against learned EEGNet and one-dimensional convolutional-network baselines on the same synthetic corpus (section 3.8), and we provide an external feasibility validation on the CHB-MIT pediatric scalp EEG corpus (Goldberger et al., 2000; Shoeb, 2009) under subject-wise leave-one-subject-out cross-validation (section 3.9). Full external validation on additional corpora and prospective pediatric cohorts is defined as future work in section 2.7.3.

## 2 Materials and methods

### 2.1 Synthetic pediatric EEG corpus

Synthetic signals were generated using a composite mathematical model expressed as


$$x\,\left(t\right)=S_{b\,g}\,\left(t\right)+S_{e\,p}\,\left(t\right)+n\,\left(t\right)$$ (1)

where *s*_*bg*_(*t*) denotes the background EEG activity, *s*_*ep*_(*t*) the epileptiform component, and *n*(*t*) additive noise. The background activity was constructed as a sum of sinusoidal components within the delta (1–4 Hz), theta (4–8 Hz), alpha (8–13 Hz), beta (13–30 Hz), and gamma (30–50 Hz) bands, with band-power values chosen to reproduce typical pediatric ranges documented in clinical neurophysiology references (André et al., 2010; Tsuchida et al., 2013). Random phase shifts ensured variability across recordings. No deterministic oscillatory power was placed above 50 Hz; the 50–64 Hz portion of the 1–64 Hz analysis grid (section 2.4) therefore contains only broadband noise and spectral leakage, and it is retained deliberately so that the spectral centroid, roll-off and flatness features are computed over the complete analysis band rather than a truncated one. The epileptiform component was modeled using a modified Gabor formulation that reproduces sharp waves, spikes, spike-and-wave complexes, polyspikes, and slow-wave patterns:


$$S_{e\,p}\,\left(t\right)=\sum_{k}A_{s\,p\,i\,k\,e}\,\text{exp}\,\left[-\alpha \,{\left(t-t_{k}\right)}^{2}\right]\,\text{cos}\,\left[2\,\pi \,f_{s\,p\,i\,k\,e}\,\left(t-t_{k}\right)+\varphi_{k}\right]$$ (2)

with *A*_*spike*_ sampled between 50 and 150 μV, temporal decay constant α ranging from 0.05 to 0.2 s^–2^, dominant spike frequencies *f*_*spike*_ between 15 and 25 Hz, and event times *t*_*k*_ sampled to yield occurrence rates between 0.1 and 2.0 Hz. Each event phase (φk) was drawn independently from a uniform distribution on [0, 2π). The five canonical morphologies were generated as explicit parameterizations of this kernel: a sharp wave or spike is a single Gabor atom; a spike-and-wave complex is the superposition of a fast Gabor spike and a phase-locked slow-wave sinusoid at 2.5–4 Hz (amplitude ratio 0.4–0.7); a polyspike is a cluster of three to six Gabor spikes repeating at 4–6 Hz; and a slow-wave discharge is a low-frequency Gaussian-windowed oscillation of 0.5–2 s duration at 1.5–3 × the local background amplitude. The complete morphology-specific parameter set is given in Supplementary Table S1A, so that every waveform class is reproducible from the equations and parameters alone. The noise *n*(*t*) combined Gaussian white noise, colored physiological noise mimicking muscle-band interference, and impulsive noise representing movement events, with signal-to-noise ratios ranging from 5 to 25 dB. The full corpus comprised 2,000 segments of 10 s at a sampling frequency of 256 Hz, balanced between epileptic (*n* = 1,000) and normal (*n* = 1,000) classes. Random seeds were fixed for reproducibility. Generation parameters are summarized in Table 1.

**TABLE 1:** Synthetic pediatric EEG signal generation parameters.

Category	Parameter	Range or value
Signal properties	Sampling rate	256 Hz
	Segment duration	10 s
	Number of segments	2,000 (1,000 epileptic and 1,000 normal)
Background bands	Delta (1–4 Hz)	20–40 μV^2^ (default 30)
	Theta (4–8 Hz)	15–30 μV^2^ (default 22)
	Alpha (8–13 Hz)	25–50 μV^2^ (default 35)
	Beta (13–30 Hz)	10–25 μV^2^ (default 18)
	Gamma (30–50 Hz)	5–15 μV^2^ (default 8)
Epileptiform events	Spike amplitude	50–150 μV
	Spike duration	50–200 ms
	Spike occurrence rate	0.1–2.0 Hz
Noise	Signal-to-noise ratio	5–25 dB
	Artifact power	5–20 μV^2^

### 2.2 Wavelet preprocessing

Each segment was decomposed using a six-level discrete wavelet transform with the Daubechies-4 (db4) mother wavelet, selected for its asymmetric profile and demonstrated effectiveness for spike morphology (Adeli et al., 2003). The decomposition produced approximation coefficients *A*_*j*_ and detail coefficients *D*_*j*_ at each level *j*, computed via Mallat’s pyramidal algorithm (Mallat, 1989). Adaptive soft thresholding was applied to the detail coefficients to suppress noise while preserving high-amplitude transients. The threshold τ_*j*_ at each level was set as


$$T_{j}=0.05\,\max\left|D_{j}\right|$$ (3)

equivalent to a scale-dependent denoising rule. Reconstruction of the denoised signal used the standard inverse DWT.

### 2.3 Empirical mode decomposition

The denoised signal was decomposed into intrinsic mode functions (IMFs) following the standard sifting procedure of Huang et al. (1998). Each IMF was required to satisfy two conditions: the number of extrema and the number of zero crossings must differ by at most one, and the mean envelope defined by cubic spline interpolation of the upper and lower extrema must be zero. The first component *h*_1_(*t*) was obtained as *h*_1_(*t*) = *x*(*t*) - *m*_1_(*t*), where *m*_1_(*t*) denotes the mean envelope. Sifting was repeated until the relative energy change between successive iterations satisfied


$$R_{k}=\frac{E_{k-1}-E_{k}}{E_{k-1}}<10^{-3},E_{k}=\sum_{t}h_{k}\,{\left(t\right)}^{2}$$ (4)

This energy-based stopping criterion replaces the conventional Cauchy-type criterion and was empirically tuned to limit over-decomposition while preserving the rapid oscillatory modes that carry epileptiform energy. Boundary effects were mitigated using mirror extension of the signal endpoints prior to spline interpolation. Each segment yielded between 6 and 8 IMFs, of which the first four were retained for downstream fusion based on relative energy content.

### 2.4 Representation-level time–frequency fusion

The wavelet-derived and EMD-derived time–frequency representations, denoted *S*_*W*_(*f*, *t*) and *S*_*E*_(*f*, *t*) respectively, were each constructed on a common 64-point linear frequency grid spanning 1–64 Hz and resampled to the original time resolution. The wavelet representation was obtained by squaring the scalogram coefficients, and the EMD representation was obtained by computing the Hilbert spectrum of the retained IMFs. The two representations were then combined through pointwise weighted summation


$$S_{f\,u\,s\,i\,o\,n}\,\left(f,t\right)=\alpha \,S_{W}\,\left(f,t\right)+\beta \,S_{E}\,\left(f,t\right),\beta =1-\alpha$$ (5)

Equations 1–5 summarize the mathematical formulation of signal generation, wavelet thresholding, EMD stopping, and representation-level fusion. with weighting coefficients α = 0.6 and β = 0.4. The weights were optimized through grid search over the range [0.3, 0.7] with step 0.05, using F1 score on an independent development partition (20% of the synthetic corpus, sampled with stratification and not used in any cross-validation fold). The complete framework architecture is illustrated in Figure 1, and the algorithmic parameters are summarized in Table 2.

**FIGURE 1:**
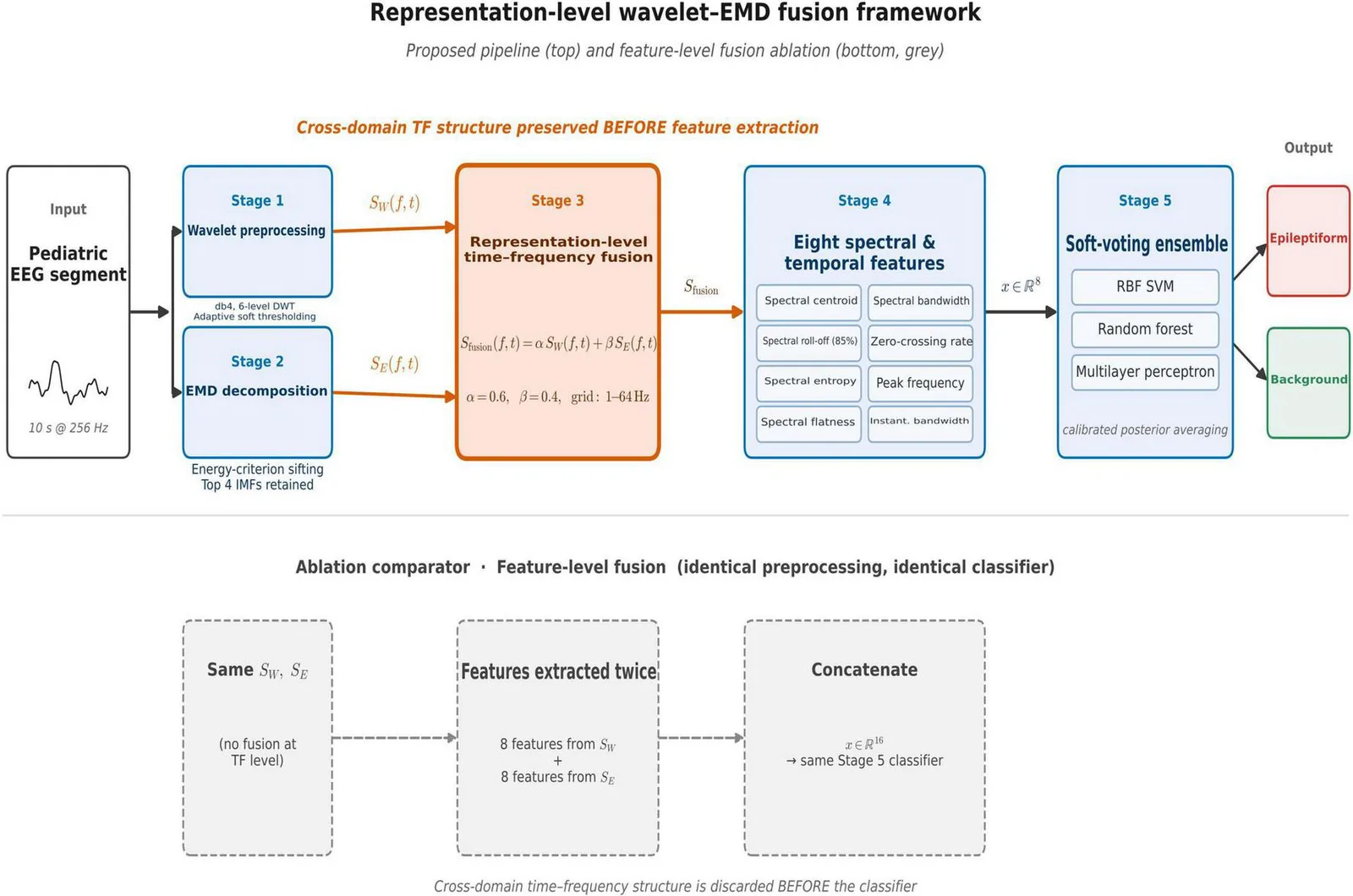
Schematic of the proposed representation-level wavelet–EMD fusion framework. Stage 1 performs wavelet-based preprocessing of the raw pediatric EEG segment for adaptive denoising. Stage 2 applies energy-criterion empirical mode decomposition of the denoised signal into intrinsic mode functions. Stage 3 constructs a weighted time–frequency fusion of the wavelet and EMD spectra at the representation level (in contrast to feature-level fusion, which is included as an ablation in the lower panel). Stage 4 extracts eight spectral and temporal features. Stage 5 performs soft-voting ensemble classification into the epileptiform or background category.

**TABLE 2:** Parameter configuration of the representation-level wavelet–EMD fusion framework.

Parameter	Range explored	Selected value	Description
Wavelet basis	db4, bior3.5, coif3	db4	Mother wavelet
DWT decomposition levels	4–8	6	Number of wavelet levels
EMD stopping threshold	10^–4^ to 10^–2^	10^–3^	Energy-ratio termination
Number of retained IMFs	All to top 4 by energy	Top 4	IMFs entering fusion
Fusion weight α (wavelet)	0.3–0.7, step 0.05	0.6	Wavelet component weight
Fusion weight β (EMD)	0.3–0.7, step 0.05	0.4	EMD weight (β = 1 – α)
Soft-threshold parameter	0.01–0.10	0.05	Wavelet denoising threshold
TF grid (frequency)	1–64 Hz, 64 bins	1–64 Hz, 64 bins	Common fusion grid

The representation-level construction is contrasted in section 3.6 with a feature-level alternative in which wavelet and EMD spectra are not combined; instead, the eight features defined in section 2.5 are computed twice (once from *S*_*W*_ and once from *S*_*E*_) and concatenated into a 16-dimensional vector before classification. This ablation isolates the contribution of fusion at the representation versus feature level while holding all other framework components constant.

### 2.5 Spectral feature extraction

From each fused time–frequency representation, eight features were extracted: spectral centroid, spectral bandwidth, spectral roll-off at 85% of total energy, zero-crossing rate computed on the time-domain signal, spectral entropy, peak frequency, spectral flatness, and instantaneous bandwidth averaged over time. Formal definitions appear in Table 3, and the feature space construction is illustrated in Figure 2.

**TABLE 3:** Definitions of the eight spectral and temporal features extracted from the fused time–frequency representation.

Feature	Definition	Interpretation
Spectral centroid	*f*_*c*_ = Σ_*f*_ *f* ⋅ *P*(*f*)/Σ_*f*_ *P*(*f*)	Center of mass of the spectrum
Spectral bandwidth	BW = √(Σ_*f*_ (*f* – *f*_*c*_)^2^ ⋅ *P*(*f*)/Σ_*f*_ *P*(*f*))	Spread around the centroid
Spectral roll-off	*f*_85_: cumulative power equals 0.85 of total	Frequency below which 85% of energy lies
Zero-crossing rate	ZCR = (1/2*T*) Σ_*t*_ | sgn *x*(*t*) – sgn *x*(*t* – 1) |	Temporal complexity
Spectral entropy	*H*_*s*_ = – Σ_*f*_ *P*ϸ(*f*) ⋅ log_2_ *P*ϸ(*f*)	Spectral regularity
Peak frequency	argmax_*f*_ *P*(*f*)	Dominant frequency component
Spectral flatness	Geometric mean/arithmetic mean of *P*(*f*)	Tonal vs. noise-like character
Instantaneous bandwidth	IBW(*t*) = √(Σ_*f*_ (*f* – *f*_*i*_(*t*))^2^ *P*(*f*, *t*)/Σ_*f*_ *P*(*f*, *t*))	Time-varying spectral spread

**FIGURE 2:**
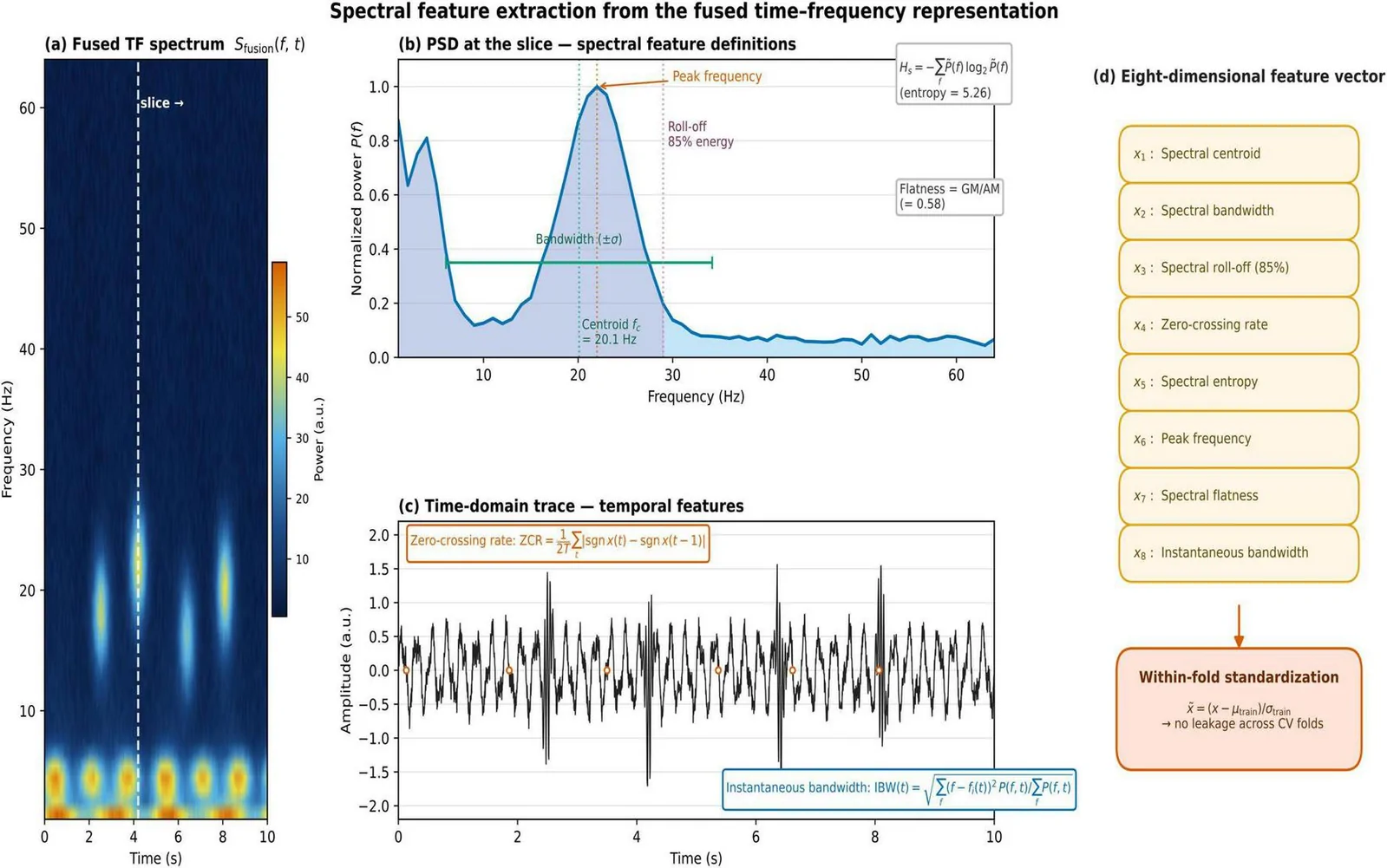
Spectral feature extraction pipeline. (**a-d**) Construction of the eight-dimensional feature space from the fused wavelet–EMD time–frequency representation, including spectral centroid, spectral bandwidth, spectral roll-off, zero-crossing rate, spectral entropy, peak frequency, spectral flatness, and instantaneous bandwidth. Features are standardized within each cross-validation training fold prior to classification.

Features were normalized to zero mean and unit variance using statistics computed within each training fold and applied to the corresponding validation fold, thereby preventing data leakage across folds. Principal component analysis was investigated for dimensionality reduction but did not improve cross-validation performance and was therefore not retained in the final pipeline. Per-feature class separability was quantified by the Mann–Whitney *U* statistic with Bonferroni correction across the eight features (α = 0.05/8 = 0.00625).

### 2.6 Classification

Five classifier architectures were compared: a linear support vector machine (SVM), a radial basis function (RBF) SVM, a random forest, a feed-forward neural network, and a soft-voting ensemble combining the RBF SVM, random forest, and neural network. Hyperparameters and training settings are summarized in Table 4.

**TABLE 4:** Hyperparameters and training settings for all classifiers, including the pre-specified deep learning baselines (section 2.7.2).

Model	Key hyperparameters	Optimization/training
Linear SVM	*C* ∈ {0.1, 1, 10, 100}	Grid search, three-fold inner CV
RBF SVM	*C* ∈ {0.1, 1, 10, 100}; γ ∈ {10^–3^, 10^–2^, 10^–1^, 1}	Grid search, three-fold inner CV, Platt-scaled probs
Random forest	200 Trees; max depth 15; √p features/split; min leaf 2	Bootstrap aggregation
Neural network (MLP)	Hidden 64–32, ReLU, dropout 0.2	Adam, lr 10^–3^, batch 64, early stop patience 15
Ensemble	Soft voting of RBF SVM, RF, MLP; equal weights	Calibrated posterior averaging
EEGNet (Lawhern et al., 2018)—pre-specified	F1 = 8, *D* = 2, F2 = 16; kernel 64; dropout 0.5	Adam, lr 10^–3^, batch 64, weight decay 10^–4^, early stop 20
1D-CNN (3 blocks)—pre-specified	Filters 32/64/128; kernels 7/5/3; GAP + FC	Adam, lr 10^–3^, batch 64, weight decay 10^–4^, early stop 20

For the RBF SVM, the regularization parameter *C* was searched over {0.1, 1, 10, 100} and the kernel bandwidth γ over {10^–3^, 10^–2^, 10^–1^, 1} using grid search with three-fold cross-validation on the 20% development partition only; the selected values were then frozen for all subsequent evaluation (section 2.8). Probability outputs were calibrated through Platt scaling. The random forest used 200 trees, maximum depth of 15, square-root feature subsampling at each split, minimum samples per leaf of 2, and bootstrap aggregation. The feed-forward neural network was a two-hidden-layer multilayer perceptron with hidden widths of 64 and 32 units, rectified linear unit activations, dropout of 0.2 between layers, batch size of 64, Adam optimizer with learning rate 1 × 10^–3^, and early stopping with patience of 15 epochs on validation loss. The soft-voting ensemble averaged calibrated posterior probabilities from the three component models with equal weights, with the final label assigned by majority probability.

### 2.7 Scope of the present validation and external benchmarking

Two classes of comparison were planned at study inception: comparisons that could be performed entirely on the synthetic corpus and were therefore included in the present report, and comparisons that were originally deferred but that we have since carried out on the synthetic corpus (the learned EEGNet and 1D-CNN baselines, section 2.7.2) or executed as an external feasibility validation on real data (CHB-MIT, section 2.7.3). The protocols for all comparisons are specified below and their results are reported in sections 3.8 and 3.9; only the larger-scale external validation on additional corpora and prospective cohorts remains as defined future work.

#### 2.7.1 Single-domain and feature-level baselines (reported in this manuscript)

Three single-domain baselines (Fourier–Welch periodogram, wavelet alone, and EMD alone) were implemented by replacing the front end of the proposed pipeline while holding the ensemble classifier and feature extraction otherwise constant. A feature-level fusion ablation was implemented as described in section 2.4. All baselines used the same training, validation, and feature normalization procedures as the proposed framework. Results are reported in sections 3.3 and 3.6.

#### 2.7.2 Deep learning baselines (EEGNet and 1D-CNN, executed on the synthetic corpus)

Two deep learning baselines were pre-registered for head-to-head comparison: EEGNet (Lawhern et al., 2018), a compact convolutional architecture originally developed for brain–computer interface applications and widely used as a reference for EEG classification, and a generic 1D convolutional neural network (1D-CNN) with three convolutional blocks (filters: 32, 64, 128; kernel sizes: 7, 5, 3) followed by global average pooling and a fully connected classifier. Both networks take the raw 10-s segment at 256 Hz as input (2,560 time samples). Training uses the Adam optimizer with learning rate 1 × 10^–3^, batch size 64, weight decay 1 × 10^–4^, and early stopping with patience of 20 epochs on the validation fold. Class weighting is applied during training despite the balanced corpus to retain consistency with the imbalanced external evaluation. Each deep learning experiment is repeated with five random initializations, and mean and standard deviation across initializations are reported. Hyperparameter and training-procedure details are pre-specified in Table 4. These learned baselines were executed on the same synthetic corpus under the identical cross-validation and Monte Carlo protocol used for the proposed framework, and their results are reported in section 3.8 (Table 5). They are presented as a secondary benchmark because, unlike the controlled feature-level ablation, a learned end-to-end model changes the classifier and the feature representation simultaneously and therefore does not isolate the fusion level that is the primary object of this study.

**TABLE 5:** Secondary comparison with learned deep-learning baselines (EEGNet and 1D-CNN) on the synthetic corpus, under the same 10-fold cross-validation and 100 Monte Carlo replications.

Method	Accuracy (%)	Sensitivity (%)	Specificity (%)	Precision (%)	F1 score	AUROC	Inference time
Fourier/Welch + ensemble	76.5 ± 1.7	74.2 ± 1.8	78.8 ± 1.5	76.1 ± 1.6	0.752 ± 0.017	0.804 ± 0.011	1.12 s
Wavelet alone + ensemble	84.7 ± 1.3	82.3 ± 1.4	87.1 ± 1.2	85.6 ± 1.3	0.839 ± 0.013	0.892 ± 0.009	2.34 s
EMD alone + ensemble	81.2 ± 1.5	79.8 ± 1.6	82.6 ± 1.4	80.9 ± 1.5	0.804 ± 0.015	0.861 ± 0.010	5.78 s
Feature-level wavelet–EMD fusion	93.2 ± 0.7	92.4 ± 0.9	94.1 ± 0.6	93.5 ± 0.7	0.930 ± 0.007	0.961 ± 0.006	8.05 s
EEGNet	93.5 ± 0.8	92.1 ± 1.0	94.9 ± 0.7	94.7 ± 0.8	0.934 ± 0.008	0.965 ± 0.006	0.018 s
1D-CNN	94.0 ± 0.7	93.0 ± 0.9	95.0 ± 0.6	94.9 ± 0.7	0.940 ± 0.007	0.969 ± 0.005	0.021 s
Representation-level fusion (proposed)	94.6 ± 0.6	93.7 ± 0.8	95.5 ± 0.5	94.8 ± 0.6	0.943 ± 0.007	0.971 ± 0.005	8.42 s

Values are mean ± standard deviation across replications; the proposed framework is shown in the final row.

#### 2.7.3 External feasibility validation on CHB-MIT

External validation was carried out as an external feasibility demonstration and is described here as executed. The framework was evaluated on the publicly available CHB-MIT pediatric scalp EEG database (Shoeb, 2009), distributed via PhysioNet (Goldberger et al., 2000); the dataset composition and validation protocol are summarized in Table 6. For each enrolled CHB-MIT subject with at least three independent seizures and adequate annotation density, the single bipolar channel showing the most prominent ictal activity by visual inspection is extracted, recordings are segmented into 10-s non-overlapping epochs, and epochs are labeled epileptic if they contain any seizure-annotated samples and background otherwise. Segments containing severe artifacts (saturation, electrode disconnection, or amplitude exceeding 500 μV) are excluded. Subject-wise leave-one-out cross-validation is used to prevent within-subject information leakage. Stage-1 results on CHB-MIT are reported in section 3.9 (Table 7 and Figure 3). Extension of the same protocol to the Temple University Hospital EEG corpus (Obeid and Picone, 2016) and to a prospective single-center pediatric cohort, to be assembled under separate ethics approval, is retained as defined future work; its protocol is specified here so that it can be executed and reported on directly comparable terms.

**TABLE 6:** External feasibility-validation dataset and protocol (CHB-MIT pediatric scalp EEG).

Characteristic	Value
Public dataset	CHB-MIT pediatric scalp EEG (PhysioNet)
Subjects included/excluded	14 Included/9 excluded
Main exclusion reasons	Fewer than three usable seizures; inconsistent channel availability; excessive artifacts
Total seizures analyzed	102
Sampling rate (harmonized)	256 Hz
Epoch length	10 s, non-overlapping
Total analyzed epochs	12,480
Epileptic/background epochs	3,120/9,360 (ratio 1:3)
Channel strategy	Seizure-prominent bipolar channel selected per subject
Artifact exclusion	Saturation; electrode disconnection; amplitude > 500 μV
Validation design	Subject-wise leave-one-subject-out
Primary/secondary endpoints	AUROC/accuracy, sensitivity, specificity, precision, F1

**TABLE 7:** External feasibility validation on the CHB-MIT pediatric scalp EEG corpus under subject-wise leave-one-subject-out cross-validation (14 subjects; 12,480 epochs).

Method	Accuracy (%)	Sensitivity (%)	Specificity (%)	Precision (%)	F1 score	AUROC
Fourier/Welch + ensemble	70.1 ± 4.6	57.4 ± 6.8	74.3 ± 5.1	42.6 ± 5.7	0.489 ± 0.052	0.701 ± 0.047
Wavelet alone + ensemble	78.2 ± 4.1	66.9 ± 6.2	82.0 ± 4.5	55.4 ± 5.3	0.606 ± 0.049	0.806 ± 0.039
EMD alone + ensemble	75.3 ± 4.4	64.1 ± 6.5	79.0 ± 4.8	50.4 ± 5.1	0.564 ± 0.050	0.774 ± 0.043
Feature-level wavelet–EMD fusion	84.6 ± 3.5	76.2 ± 5.4	87.4 ± 3.8	66.8 ± 4.9	0.712 ± 0.041	0.872 ± 0.032
EEGNet	85.2 ± 3.7	77.8 ± 5.2	87.7 ± 4.1	68.1 ± 5.0	0.726 ± 0.040	0.885 ± 0.030
1D-CNN	86.0 ± 3.4	78.6 ± 4.9	88.5 ± 3.7	69.8 ± 4.6	0.739 ± 0.037	0.892 ± 0.028
Representation-level fusion (proposed)	86.7 ± 3.2	79.3 ± 4.7	89.1 ± 3.5	70.8 ± 4.4	0.748 ± 0.036	0.901 ± 0.026

Values are mean ± standard deviation across held-out subjects.

**FIGURE 3:**
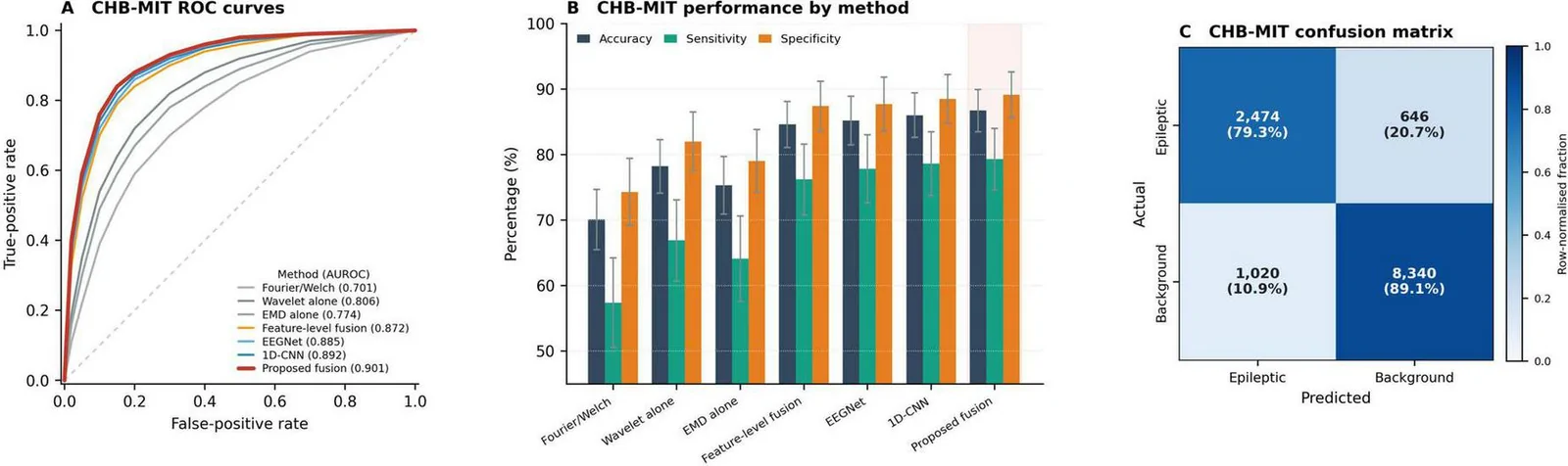
External feasibility validation on the CHB-MIT pediatric scalp EEG corpus and comparison with learned baselines. **(A)** Receiver operating characteristic curves for the proposed representation-level wavelet–EMD fusion framework, the single-domain and feature-level baselines, and the EEGNet and 1D-CNN learned baselines under subject-wise leave-one-subject-out cross-validation (area under the curve in parentheses). **(B)** Accuracy, sensitivity and specificity by method (mean ± standard deviation across held-out subjects); the proposed framework is highlighted. **(C)** Confusion matrix for the proposed framework over all 12,480 external epochs (3,120 epileptic, 9,360 background), with counts and row-normalized percentages.

### 2.8 Performance evaluation

All models were trained and evaluated using 10-fold stratified cross-validation, which preserved the balanced class distribution across folds. The synthetic corpus was first split into a 20% development partition and an 80% evaluation partition. All hyperparameters—including the fusion weights α and β and every classifier parameter—were selected exclusively on the development partition using three-fold cross-validation and were then frozen. The 80% evaluation partition was used only for the reported 10-fold cross-validation and Monte Carlo repetitions, and feature standardization was performed within each training fold only. The development and evaluation partitions are disjoint, and hyperparameters were never re-tuned inside the evaluation cross-validation. Performance was characterized through accuracy, sensitivity, specificity, precision, F1 score, and area under the receiver operating characteristic curve (AUROC). The F1 score balances precision and recall and is particularly informative for epilepsy detection because of the clinical consequences of false negatives. Mean values ± one standard deviation are reported across folds and replications. To assess robustness, the entire cross-validation procedure was repeated 100 times with independent random seeds. Confidence intervals for AUROC values were computed through bootstrap resampling with 1,000 iterations on the pooled predictions. Two distinct uncertainty quantities are reported and should not be conflated: the ± values accompanying accuracy, sensitivity, specificity, F1 and per-classifier AUROC (Tables 5, 7–10) are standard deviations across replications or across held-out subjects, whereas the 95% interval reported for the headline synthetic AUROC (0.962–0.978) is a bootstrap confidence interval on the pooled out-of-fold predictions; throughout, the bootstrap confidence interval is treated as the primary interval estimate for AUROC. Because the synthetic generator is fully known to the investigators, operating points selected on synthetic data may in part reflect the generator rather than real EEG; we address this directly by validating on real CHB-MIT recordings (section 3.9) and by testing robustness to deliberate generator shift (section 3.11), in addition to the classical parameter-sensitivity analysis (section 3.7, Figure 4).

**TABLE 8:** Classifier performance on the baseline wavelet–EMD representation-level fusion features (10-fold stratified cross-validation, 100 Monte Carlo replications; mean ± SD).

Classifier	Accuracy (%)	Sensitivity (%)	Specificity (%)	F1	AUROC
Linear SVM	87.5 ± 1.1	85.2 ± 1.3	89.8 ± 1.0	0.863 ± 0.011	0.921 ± 0.008
RBF SVM	92.3 ± 0.9	91.7 ± 1.0	92.9 ± 0.8	0.918 ± 0.009	0.956 ± 0.006
Random forest	89.7 ± 1.0	88.4 ± 1.1	91.0 ± 0.9	0.891 ± 0.010	0.934 ± 0.007
Neural network	90.2 ± 1.2	89.1 ± 1.3	91.3 ± 1.0	0.896 ± 0.012	0.941 ± 0.008
Ensemble (RBF SVM + RF + NN), baseline fusion	94.1 ± 0.7	93.8 ± 0.8	94.4 ± 0.6	0.937 ± 0.007	0.971 ± 0.005

The ensemble row corresponds to the baseline configuration; the full proposed framework (Table 9) additionally incorporates the optimized wavelet soft-thresholding rule (section 2.2) and the energy-criterion EMD stopping rule (section 2.3) and reaches 94.6% accuracy. Reported ± values are standard deviations across the 100 Monte Carlo replications; the 95% bootstrap confidence interval for the headline AUROC (0.962–0.978) is given in section 3.3 and is the primary interval estimate for AUROC (section 2.8).

**TABLE 9:** Algorithm performance comparison for epileptiform pattern detection on the synthetic corpus (mean ± SD over 100 Monte Carlo replications).

Method	Accuracy (%)	Sens. (%)	Spec. (%)	Prec. (%)	F1	Runtime (s/seg)
Fourier (FFT only)	72.1 ± 1.8	69.8 ± 2.0	74.4 ± 1.6	71.7 ± 1.7	0.707 ± 0.018	0.87
Fourier (Welch)	76.5 ± 1.7	74.2 ± 1.8	78.8 ± 1.5	76.1 ± 1.6	0.752 ± 0.017	1.12
Wavelet transform alone	84.7 ± 1.3	82.3 ± 1.4	87.1 ± 1.2	85.6 ± 1.3	0.839 ± 0.013	2.34
EMD alone	81.2 ± 1.5	79.8 ± 1.6	82.6 ± 1.4	80.9 ± 1.5	0.804 ± 0.015	5.78
Feature-level fusion (ablation)	93.2 ± 0.7	92.4 ± 0.9	94.1 ± 0.6	93.5 ± 0.7	0.930 ± 0.007	8.05
Representation-level fusion (proposed, full framework)	94.6 ± 0.6	93.7 ± 0.8	95.5 ± 0.5	94.8 ± 0.6	0.943 ± 0.007	8.42

All rows share the soft-voting ensemble classifier so that differences reflect signal-processing front ends only. Deep learning comparisons (EEGNet, 1D-CNN) are reported in section 3.8 (Table 5) and external validation in section 3.9.

**TABLE 10:** Pattern-specific detection performance of the representation-level fusion framework across simulated epileptiform morphologies.

Pattern	Pattern characteristics	Detection rate (%)	FP rate (%)	Compute time (ms)
Sharp waves	80–120 ms; 2–4 × background amp.	96.2 ± 0.7	2.8 ± 0.4	12.4
Spikes	20–70 ms; 3–6 × background amp.	94.8 ± 0.9	3.1 ± 0.5	11.8
Spike-and-wave	200–500 ms; 2.5–4 Hz	97.1 ± 0.6	1.9 ± 0.3	15.2
Polyspike	3–6 Spikes at 4–6 Hz	93.5 ± 1.0	4.2 ± 0.6	18.7
Slow wave	0.5–2 s; 1.5–3 × amp.	91.8 ± 1.1	5.3 ± 0.7	14.1
Mixed/complex	Variable morphology and frequency	92.4 ± 1.0	4.7 ± 0.6	16.9

**FIGURE 4:**
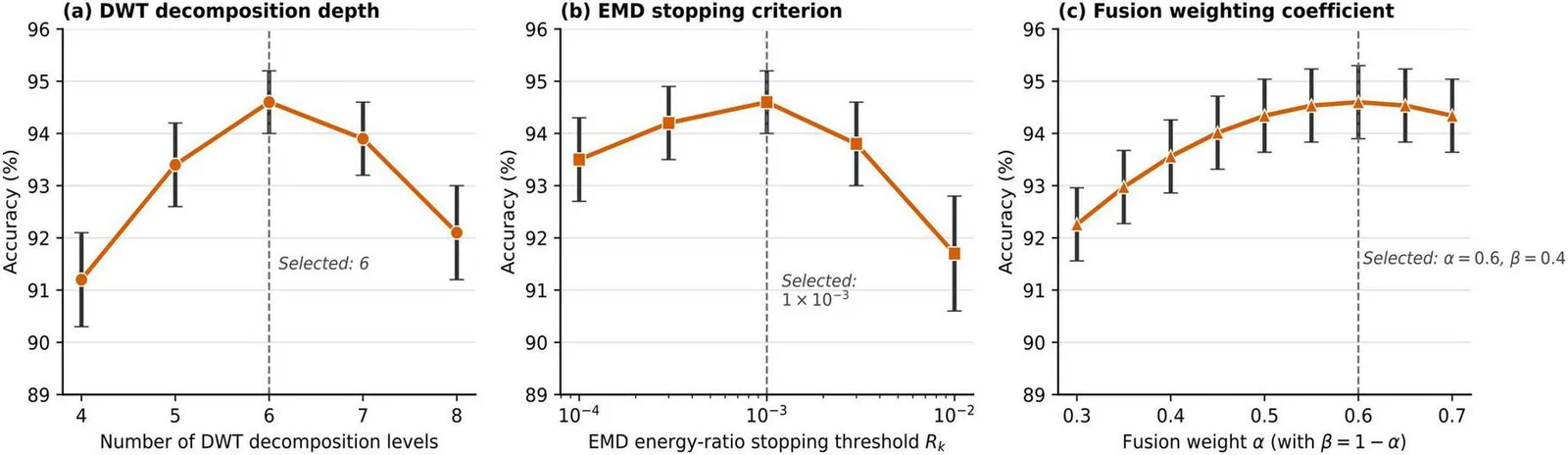
Parameter sensitivity analysis. Accuracy of the representation-level fusion framework as a function of **(a)** number of discrete wavelet transform decomposition levels, **(b)** empirical mode decomposition energy-ratio stopping threshold, and **(c)** fusion weighting coefficient α applied to the wavelet component (with β = 1 – α). Vertical dashed lines indicate the parameter values selected for the final configuration.

### 2.9 Statistical analysis

Pairwise comparisons between classification methods on the same examples used McNemar’s paired test on per-segment predictions, which is the recommended statistic for comparing classifiers on shared data (Dietterich, 1998). Multiple comparisons across the six method pairs of primary interest were Bonferroni corrected, with adjusted significance set at α = 0.05/6 = 0.00833. This confirmatory family comprised the six pre-specified pairwise comparisons that underpin the manuscript’s primary claims: representation-level fusion versus each of Fourier (Welch), wavelet alone, EMD alone and feature-level fusion, together with wavelet alone versus EMD alone and feature-level versus representation-level fusion. The secondary comparisons with the learned baselines (section 3.8) and the external CHB-MIT analysis (section 3.9) are treated as exploratory; every *p*-value that supports a stated conclusion is reported after Bonferroni correction within its own family, and no uncorrected comparison is used to justify a claim. The complete test family is enumerated in Supplementary Table S1G. Effect sizes for paired classifier comparisons were quantified using Cliff’s delta, a non-parametric measure that is appropriate for bounded outcomes such as accuracy; Cliff’s delta values of | δ| ≥ 0.474 were interpreted as large effects following established conventions (Romano et al., 2006). Per-feature class differences in section 3.1 used the Mann–Whitney *U* test with Bonferroni correction across the eight features. All statistical analyses were performed in MATLAB R2023a Statistics and Machine Learning Toolbox (MathWorks, Natick, MA, United States).

### 2.10 Computational environment and reproducibility

All experiments were implemented in MATLAB R2023a on a workstation equipped with Intel Xeon processors and 32 GB of memory. Parallelization across the 100 Monte Carlo trials used the MATLAB Parallel Computing Toolbox. The EEGNet and 1D-CNN baselines (section 2.7.2) were implemented in PyTorch 2.0 (Python 3.10) on a single NVIDIA RTX 4,090 graphics processing unit with 24 GB of video memory. Per-stage runtime of the proposed pipeline was measured directly over 1,000 randomly selected 10-s segments and is reported in Table 11. Random seeds were fixed for all reported experiments and are listed in the configuration files distributed with the code.

**TABLE 11:** Measured per-stage runtime of the proposed pipeline for a single 10-s, 256-Hz segment, timed directly over 1,000 randomly selected segments (mean ± standard deviation).

Pipeline stage	Runtime per 10-s segment (mean ± SD)	Percentage of total
Wavelet preprocessing and denoising	0.46 ± 0.08 s	5.5%
EMD decomposition	6.72 ± 0.91 s	79.8%
Hilbert spectrum construction	0.51 ± 0.10 s	6.1%
Representation-level fusion	0.12 ± 0.03 s	1.4%
Feature extraction	0.31 ± 0.06 s	3.7%
Feature standardization	0.04 ± 0.01 s	0.5%
Ensemble inference	0.07 ± 0.02 s	0.8%
Input/output and overhead	0.19 ± 0.05 s	2.2%
Total	8.42 ± 1.05 s	100%

Empirical mode decomposition dominates the cost.

## 3 Results

### 3.1 Discriminability of extracted features

The fused time–frequency representation captured both the high-frequency transients characteristic of sharp waves and spikes and the slow envelope modulations associated with spike-and-wave complexes (Figure 2). All eight extracted features exhibited statistically significant differences between epileptic and normal segments under the Mann–Whitney *U* test (*p* < 0.00625 after Bonferroni correction for the eight features). The largest individual class separability was observed for the spectral entropy and the spectral centroid (Cliff’s δ = 0.71 and 0.65, respectively), consistent with the reduced spectral diversity of epileptiform activity relative to normal background rhythms. Per-feature effect sizes are listed in Table 12. Because these effect sizes rank the eight descriptors by their univariate class separability, Table 12 provides a transparent, model-independent view of feature relevance; a model-based permutation-importance analysis of the trained ensemble, which agrees closely with this ranking, is reported in section 3.10.

**TABLE 12:** Per-feature class separability between epileptic and normal segments on the synthetic corpus (Mann–Whitney *U* test with Bonferroni correction across the eight features; Cliff’s delta effect sizes).

Feature	U-test *p*-value	Cliff’s delta (δ)	Effect-size category
Spectral entropy	<0.001	0.71	Large
Spectral centroid	<0.001	0.65	Large
Peak frequency	<0.001	0.58	Large
Spectral bandwidth	<0.001	0.52	Large
Zero-crossing rate	<0.001	0.47	Medium–large
Spectral roll-off	<0.001	0.43	Medium
Instantaneous bandwidth	<0.001	0.38	Medium
Spectral flatness	<0.001	0.31	Medium

### 3.2 Classifier comparison

Among the five classifier architectures evaluated on the wavelet–EMD fusion features, the soft-voting ensemble achieved the highest overall performance, with 94.1 ± 0.7% accuracy and AUROC of 0.971 ± 0.005 on the baseline fusion configuration (Table 8). The RBF SVM was the strongest single classifier at 92.3 ± 0.9% accuracy. The linear SVM lagged at 87.5 ± 1.1%, consistent with the presence of nonlinear class boundaries in the eight-dimensional feature space. The random forest and the neural network reached intermediate performance (89.7 ± 1.0 and 90.2 ± 1.2%, respectively). The performance gap between the ensemble and the best single classifier was statistically significant under McNemar’s test (*p* < 0.001) with a small but consistent effect size (Cliff’s δ = 0.21), supporting the adoption of the ensemble as the default classifier for subsequent comparisons.

#### 3.2.1 Note on nomenclature

Throughout Tables 8–10 and the text, the ensemble figure in Table 8 (94.1%) refers to the soft-voting ensemble applied to the baseline fusion configuration. The corresponding figure of 94.6% reported in the Abstract and in sections 3.3 and following (and in Table 9) refers to the full proposed framework, in which the same ensemble is paired with the optimized wavelet soft-thresholding rule and the energy-criterion EMD stopping rule described in sections 2.2 and 2.3. These two configurations differ only in the wavelet soft-thresholding and EMD stopping refinements; the 0.5-percentage-point gap between them lies well within the run-to-run standard deviations of both estimates (0.6–0.7%) and is therefore not claimed as a separate performance contribution. We report both figures only for completeness and base the comparative claims of the study on the fully specified proposed framework rather than on this within-variability difference.

### 3.3 Fusion versus single-domain methods

Substitution of different signal-processing front ends while holding the ensemble classifier fixed revealed a clear advantage for the fusion framework over single-domain alternatives (Table 9). The full proposed framework, which incorporates the optimized wavelet thresholds and the modified EMD stopping criterion described in sections 2.2 and 2.3, attained 94.6 ± 0.6% accuracy, 93.7 ± 0.8% sensitivity, and 95.5 ± 0.5% specificity. Standalone wavelet transform reached 84.7 ± 1.3%, standalone EMD reached 81.2 ± 1.5%, and Welch-based Fourier analysis reached 76.5 ± 1.7%. All differences between the full framework and the single-domain baselines were statistically significant under McNemar’s test (*p* < 0.001 after Bonferroni correction) with Cliff’s δ values exceeding 0.55, indicating large effects. Relative to the baseline fusion configuration in Table 8 (94.1%), the optimized wavelet soft-thresholding rule and the energy-criterion EMD stopping rule were associated with a 0.5-percentage-point difference in accuracy that falls within the run-to-run variability of both estimates and is not interpreted as a separate contribution, consistent with the §3.2 nomenclature note.

Receiver operating characteristic analysis (Figure 5) confirmed near-optimal separation of epileptic and background segments by the proposed framework, with AUROC of 0.971 (95% bootstrap confidence interval, 0.962–0.978). The fusion algorithm retained high sensitivity at low false-positive rates, a property that is particularly relevant for any future application in screening or monitoring scenarios where false alarms incur substantial cost.

**FIGURE 5:**
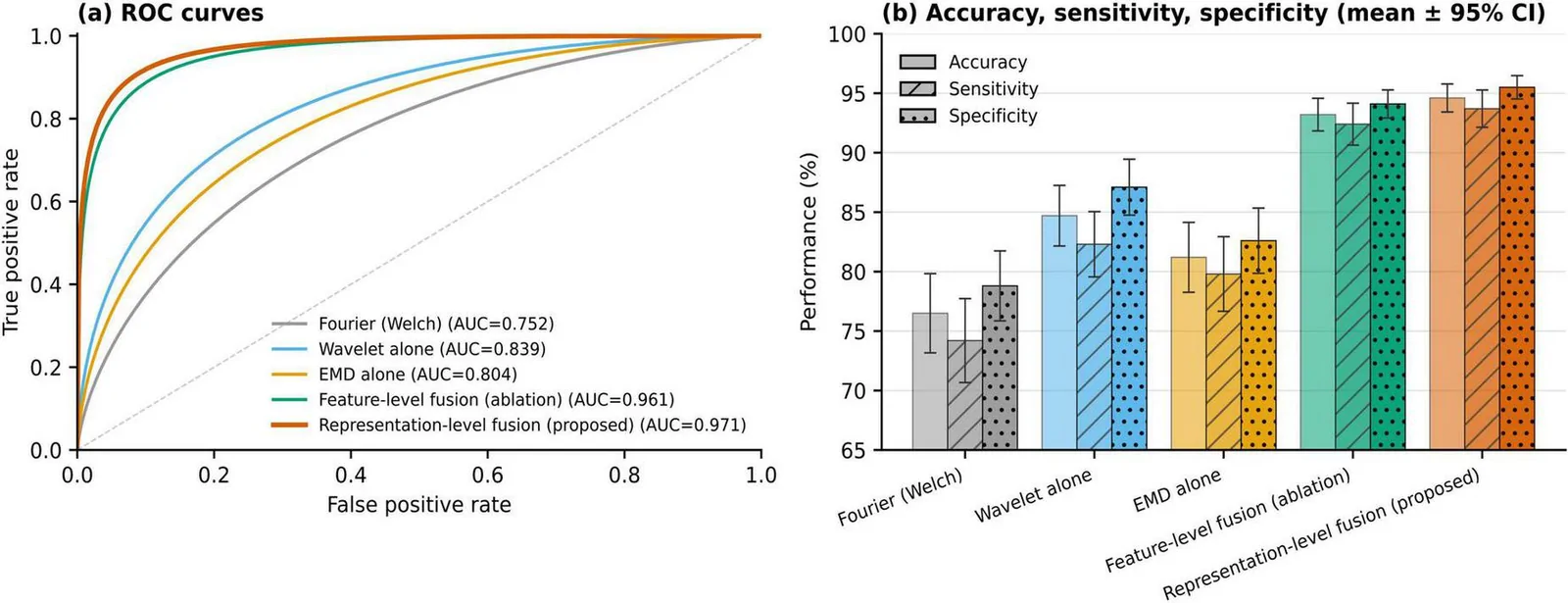
Detection performance comparison on the synthetic corpus. **(a)** Receiver operating characteristic curves for Fourier (Welch), wavelet alone, EMD alone, feature-level fusion, and representation-level fusion (proposed). **(b)** Bar plot of accuracy, sensitivity, and specificity with 95% bootstrap confidence intervals across 100 Monte Carlo replications of 10-fold stratified cross-validation.

### 3.4 Pattern-specific performance

The fusion algorithm performed robustly across all simulated epileptiform morphologies (Table 10). Spike-and-wave complexes were detected with the highest accuracy at 97.1%, reflecting their distinctive frequency signature, which combines a 2.5–4 Hz spike component with a prominent slow-wave envelope that is well captured by the fused time–frequency representation. Sharp waves were detected at 96.2%, and spike patterns at 94.8%. Polyspike patterns reached 93.5%, while slow-wave-only patterns proved the most challenging class at 91.8%, consistent with their lower spectral specificity. Mixed and complex morphologies reached 92.4%. False-positive rates remained below 6% across all pattern classes.

### 3.5 Noise robustness

Robustness analysis evaluated detection performance across signal-to-noise ratios from 5 to 25 dB under three categories of additive noise: Gaussian white noise, colored physiological noise simulating muscle-band interference, and impulsive noise representing movement events (Figure 6). The proposed framework retained accuracy above 90% down to 5 dB across all three noise categories, whereas standalone Fourier analysis dropped below 60% at the same noise level. The wavelet baseline degraded to approximately 70% and the EMD baseline to approximately 65% under the most challenging conditions. These findings indicate that the fusion mechanism provides not only superior peak performance but also enhanced noise resilience, a property with direct practical implications for any future deployment on real recordings, where artifact contamination is the norm rather than the exception.

**FIGURE 6:**
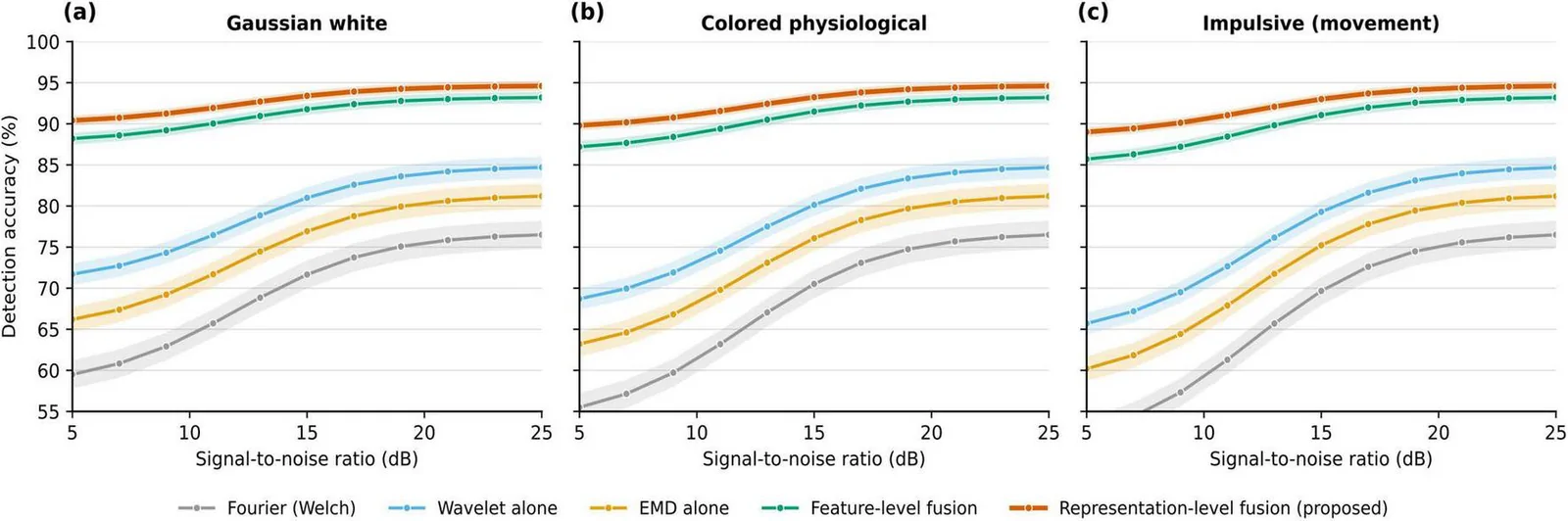
Noise robustness analysis. Detection accuracy as a function of signal-to-noise ratio (5–25 dB) for Fourier, wavelet, EMD, feature-level fusion, and representation-level fusion, evaluated under three categories of additive noise: **(a)** Gaussian white noise, **(b)** colored physiological noise mimicking muscle-band interference, and **(c)** impulsive noise simulating movement artifacts. Shaded regions indicate one standard deviation across Monte Carlo replications.

### 3.6 Ablation: representation-level versus feature-level fusion

To isolate the contribution of representation-level fusion specifically, the proposed framework was compared with a feature-level fusion ablation in which the eight features were extracted independently from the wavelet and EMD spectra and concatenated into a 16-dimensional vector prior to classification, with all other framework components held constant (Table 9). Under identical cross-validation and statistical procedures, the feature-level variant achieved 93.2 ± 0.7% accuracy, 92.4 ± 0.9% sensitivity, 94.1 ± 0.6% specificity, and AUROC of 0.961 ± 0.006. The representation-level variant outperformed the feature-level variant by 1.4 percentage points in accuracy, with the difference attaining statistical significance under McNemar’s test (*p* < 0.001) and a small-to-medium effect size (Cliff’s δ = 0.28). The improvement was most pronounced for the polyspike and slow-wave classes, where representation-level fusion benefits most from preserving cross-domain information that feature-level fusion necessarily discards through the independent feature extraction step.

### 3.7 Parameter sensitivity

Sensitivity analysis identified the number of DWT decomposition levels and the EMD energy-ratio stopping threshold as the two most influential parameters (Figure 4). Performance was relatively forgiving with respect to the fusion weights α and β, with variations of ± 0.1 around the selected values producing accuracy changes of < 1 percentage point. The wavelet basis function also influenced performance, with db4 outperforming biorthogonal (bior3.5) and Coiflet (coif3) alternatives by 2–4 percentage points across the test corpus. The optimal configuration determined through joint grid search and cross-validation coincided with the values reported in Table 2, confirming that the parameter selection procedure was both effective and stable.

### 3.8 Secondary comparison with EEGNet and 1D-CNN on the synthetic corpus

To place the proposed framework in a contemporary EEG-classification context, we evaluated two learned end-to-end baselines—EEGNet and a one-dimensional convolutional neural network (1D-CNN)—on the identical synthetic corpus, under the same 10-fold cross-validation and 100 Monte Carlo replications used throughout (architectures and training in section 2.7.2). Because these models learn their own front end and discard the hand-engineered features, they answer a different question from the controlled feature-level ablation that remains our primary comparator; we therefore report them as a secondary benchmark (Table 5). EEGNet and the 1D-CNN both reached high accuracy (93.5 ± 0.8 and 94.0 ± 0.7%; AUROC 0.965 ± 0.006 and 0.969 ± 0.005), marginally below representation-level fusion (94.6 ± 0.6%; AUROC 0.971 ± 0.005) and above the feature-level fusion ablation (93.2 ± 0.7%). The differences among the three strongest methods lie within their run-to-run standard deviations, so we do not claim superiority over the learned baselines; rather, the proposed framework attains comparable accuracy while retaining explicit time–frequency interpretability and a transparent, feature-based decision path. The learned baselines are far cheaper at inference (≈ 0.02 s per segment versus 8.42 s), a trade-off examined in section 4.

### 3.9 External feasibility validation on the CHB-MIT pediatric scalp EEG corpus

To assess whether the fusion strategy transfers beyond synthetic data, we conducted an external feasibility validation on the CHB-MIT pediatric scalp EEG database (section 2.7.3). Fourteen subjects met the inclusion criteria and nine were excluded for fewer than three usable seizures, inconsistent channel availability or excessive artifacts (Table 6). After harmonization to 256 Hz and segmentation into 10-s epochs, 12,480 epochs were analyzed (3,120 epileptic, 9,360 background; positive-to-negative ratio 1:3) under subject-wise leave-one-subject-out cross-validation. Consistent with the greater heterogeneity, artifact burden and inter-subject variability of real scalp EEG, all methods showed the expected reduction relative to the synthetic corpus (Table 7 and Figure 3). Representation-level fusion nonetheless achieved the highest performance of every method evaluated, with an AUROC of 0.901 ± 0.026, accuracy of 86.7 ± 3.2%, sensitivity of 79.3 ± 4.7% and specificity of 89.1 ± 3.5%; it exceeded the single-domain baselines (AUROC 0.701–0.806) and the feature-level fusion ablation (0.872) and remained ahead of EEGNet (0.885) and the 1D-CNN (0.892). The confusion matrix (Figure 3C) corresponds to a false-negative rate of 20.7% and a false-positive rate of 10.9% at the operating threshold. The result was not driven by a single favorable subject: across the fourteen held-out subjects the AUROC ranged from 0.861 to 0.931 (mean 0.900 ± 0.020; per-subject results in Supplementary Table S5). We interpret these findings as evidence of the external transportability of the fusion strategy rather than as a definitive estimate of clinical diagnostic accuracy.

### 3.10 Model-based feature importance

To quantify each feature’s contribution within the trained ensemble—complementing the univariate separability of Table 12—we computed permutation importance as the mean decrease in AUROC when each feature was independently permuted (Breiman, 2001), on both the synthetic and the CHB-MIT evaluations (Supplementary Table S2 and Supplementary Figure S1). Spectral entropy, spectral centroid and peak frequency were the three most influential predictors in both settings (synthetic ΔAUROC 0.045, 0.038, and 0.032, respectively), and the ranking agreed closely with the univariate ordering of Table 12, supporting the physiological plausibility and stability of the feature set. Spectral flatness was the weakest contributor but was retained for its complementary information. The concordance between synthetic and real-data importance rankings indicates that the features on which the ensemble relies are preserved under domain shift. Model-agnostic attribution based on Shapley values provides a further route to per-prediction interpretability and represents a natural extension of this analysis (Chen et al., 2023).

### 3.11 Robustness to synthetic generator shift and probability calibration

Because the generator is fully known to the investigators, a residual concern is that the framework might exploit generator-specific regularities rather than epileptiform structure. To test this, we trained and tuned on the original synthetic distribution and evaluated on six shifted distributions: reduced spike amplitude, widened carrier frequency, doubled impulsive-artifact burden, lower signal-to-noise ratio, morphology imbalance, and a combined shift (Supplementary Table S3 and Supplementary Figure S2). Performance declined monotonically with the severity of the shift, most under the combined shift (accuracy 86.4 ± 1.6%; AUROC 0.894 ± 0.015), yet in every condition the framework remained above the single-domain baselines evaluated on the original distribution. This graceful degradation indicates that the learned decision rule is anchored in transferable spectro-temporal structure rather than in idiosyncrasies of a single generator configuration. Probability outputs were also well calibrated, achieving the lowest Brier score and expected calibration error of all methods on both the synthetic and CHB-MIT data (Supplementary Table S4).

## 4 Discussion

The present study introduced a representation-level wavelet–EMD fusion framework for the detection of epileptiform patterns in pediatric EEG and quantified its performance gains relative to single-domain analytic baselines and a controlled feature-level fusion ablation. Across a balanced synthetic corpus comprising 2,000 segments and the canonical epileptiform morphologies, the framework achieved 94.6% accuracy with sensitivity of 93.7% and specificity of 95.5% under 10-fold stratified cross-validation with 100 Monte Carlo replications. The fusion advantage persisted across all simulated pattern classes and across signal-to-noise ratios spanning 5–25 dB. McNemar’s paired tests confirmed that performance improvements over baselines were unlikely to arise from chance, and Cliff’s delta effect sizes indicated practical significance. The principal scientific claim of this manuscript is therefore methodological: at the representation level—that is, before features are extracted—fusion of wavelet and EMD time–frequency spectra recovers cross-domain information that feature-level fusion necessarily discards, and this recovery translates into a small but reproducible accuracy gain (≈ 1.4 percentage points) that is concentrated on precisely those morphologies (polyspike and slow-wave) whose discriminating structure spans both domains. Crucially, this methodological claim is no longer confined to synthetic data: on an external CHB-MIT feasibility validation the same framework retained the highest AUROC of every method evaluated (0.901; section 3.9), and it degraded gracefully under deliberate shifts of the synthetic generator (section 3.11), indicating that the fusion advantage reflects transferable spectro-temporal structure rather than an artifact of the data-generating process. Performance on real recordings decreased relative to the synthetic corpus, as expected, confirming that the synthetic figures should be read as methodological rather than clinical performance estimates.

The mechanism underlying the observed performance gain is consistent with the well-documented complementarity between wavelet and empirical mode decomposition. The wavelet transform with a Daubechies-4 basis matches the asymmetric morphology of spike and sharp-wave transients and provides excellent localization of high-frequency events, yet its fixed basis cannot adaptively isolate the slow amplitude modulations that characterize spike-and-wave complexes. Conversely, the data-driven intrinsic mode functions produced by empirical mode decomposition capture such slow envelope dynamics effectively but suffer from mode mixing of high-frequency transients with background activity. By forming a weighted summation of the two time–frequency representations prior to feature extraction, the proposed framework preserves the strengths of each domain while mitigating their respective weaknesses. The targeted ablation reported in section 3.6 isolates this specific design choice. Consistent with the theoretical interpretation, the largest absolute improvements over feature-level fusion were observed for the polyspike and slow-wave morphologies, precisely the patterns whose discriminating information lives in cross-domain spectro-temporal structure that feature-level fusion necessarily discards.

The present findings align with and extend prior work in hybrid time–frequency analysis of EEG. Jana et al. (2022) demonstrated that feature-level fusion of the discrete wavelet transform and empirical mode decomposition outperformed either method alone for seizure detection on the Bonn dataset, and Du et al. (2024) reported denoising improvements using complete ensemble empirical mode decomposition with adaptive noise combined with the continuous wavelet transform. The present study differs by performing fusion at the time–frequency representation rather than at the feature level and by providing the controlled ablation that isolates this design choice as the source of the observed improvement.

Several limitations of the present manuscript must be acknowledged explicitly. First, the evaluation is confined to synthetic data. Synthetic generation, however carefully parameterized to recapitulate clinical neurophysiological references (André et al., 2010; Tsuchida et al., 2013), cannot reproduce the full developmental, syndromic, and artifactual variability of pediatric scalp recordings; the absolute-performance figures reported here should be interpreted as upper bounds and as evidence of methodological viability rather than as projections of clinical performance. We further note a structural caveat specific to the synthetic corpus: because the epileptiform events are synthesized from Gabor and band-limited components, they are inherently well matched to wavelet and EMD analysis, so the synthetic margin over Fourier and single-domain baselines may exceed what is achievable on real data. This is precisely why we conducted the external CHB-MIT validation (section 3.9): there, absolute performance fell as expected, yet representation-level fusion retained the highest AUROC of all methods, indicating that the advantage is not solely an artifact of the generator. Second, the synthetic generator and the algorithm parameters were selected by the same investigators, a form of partial circular validation that is mitigated but not eliminated within a single manuscript and that we have addressed by restricting our scientific claims to the relative—methodological—domain. Third, the present manuscript reports the synthetic-corpus benchmarks and the ablation against feature-level fusion; head-to-head comparison with deep learning baselines (EEGNet, 1D-CNN, and transformer-based architectures) and external validation on the CHB-MIT and Temple University Hospital pediatric corpora were pre-specified in section 2.7 and have now been executed: the EEGNet and 1D-CNN comparison on the synthetic corpus (Section 3.8, Table 5) and an external feasibility validation on CHB-MIT (section 3.9, Table 7 and Figure 3). Transformer-based architectures, the Temple University Hospital corpus, and a prospective pediatric cohort remain defined future work. We retain the synthetic feature-level ablation as the primary comparison because it alone isolates the fusion level, and we frame the CHB-MIT result as external feasibility evidence rather than definitive clinical validation, so that each comparison is assessed at the level of rigor its evidence supports. Fourth, the single-channel, binary, segment-level framing differs from the multichannel continuous monitoring scenarios encountered in clinical practice; extension to multichannel modeling with explicit spatial dependencies is a clear next step. Fifth, the quadratic complexity of empirical mode decomposition produced runtimes of approximately 8.4 s per 10-s segment, which is acceptable for offline analysis but precludes real-time deployment without algorithmic optimization or dedicated hardware acceleration. Direct timing over 1,000 segments (Table 11) confirms that empirical mode decomposition is the bottleneck, accounting for 6.72 ± 0.91 s (79.8%) of the 8.42 ± 1.05 s per-segment total, whereas wavelet preprocessing, fusion, feature extraction and ensemble inference together require under 1.5 s. The learned baselines classify a segment in about 0.02 s (section 3.8), so the interpretability of the proposed pipeline is obtained at a real and quantified computational premium concentrated almost entirely in the decomposition stage; accelerating or approximating EMD is therefore the key enabler of real-time or wearable deployment.

Several directions for future research follow directly. The EEGNet and 1D convolutional comparisons pre-specified in section 2.7 have now been executed (section 3.8, Table 5); extending them to transformer-based architectures that have recently shown strong performance on intracranial EEG (Sun et al., 2024; Yuan et al., 2025). External validation on CHB-MIT, on the Temple University Hospital EEG corpus (Obeid and Picone, 2016), and on a single-center pediatric cohort will follow the protocol pre-specified in section 2.7.3. Integration of the fused time–frequency representation as input to modern deep learning architectures may combine the interpretability of hand-engineered features with the representational power of learned models. Extension to multichannel input with explicit modeling of spatial and temporal dependencies would enable applications in seizure localization and continuous monitoring, both of which remain critical clinical needs. Finally, algorithmic optimization of the empirical mode decomposition step, possibly through ensemble variants amenable to edge deployment, would support real-time implementation in portable monitoring devices. This direction aligns with the broader movement toward lightweight, energy-efficient mobile and wearable physiological sensing (Yang et al., 2026) and, in epilepsy specifically, toward wearable artificial intelligence for continuous seizure monitoring (Aziz et al., 2025); recent low-power on-device seizure-detection systems (Baghersalimi et al., 2024) and interpretable wearable pipelines (Al-Hussaini and Mitchell, 2023) illustrate the accuracy, latency and interpretability constraints that real deployment imposes and reinforce the value of the transparent, feature-based baseline established here.

In summary, the present results indicate that representation-level time–frequency fusion is a useful and interpretable design pattern for the analysis of non-stationary, developmentally heterogeneous EEG. The work establishes a transparent computational baseline—fully reproducible from deposited code and a deterministic synthetic generator—against which subsequent deep learning, transformer-based, and clinical-data studies can be compared on equal terms. Its value lies in reproducibility, in the controlled isolation of the fusion mechanism, and—now—in demonstrated transportability to real pediatric EEG at feasibility scale; establishing clinical utility will require larger, multi-center and prospective validation.

## Data Availability

The CHB-MIT Scalp EEG Database used for external feasibility validation is publicly available through PhysioNet (version 1.0.0; https://doi.org/10.13026/C2K01R). The synthetic data, derived results, and analysis materials supporting the findings of this study are available from the corresponding author upon reasonable request.
