# Supplementary material for: A representation-level wavelet–EMD fusion framework for spectral analysis and detection of epileptiform patterns in pediatric EEG: computational validation with a roadmap for external benchmarking
Source: Front Neurosci. 2026 Oct 14;20:1884876. doi: 10.3389/fnins.2026.1884876 (PMC13714712; doi:10.3389/fnins.2026.1884876)
Supplement: Supplementary file 1 [file Supplementary_file_1.docx]

# Supplementary Materials

This supplementary file accompanies the main manuscript and consolidates every numerical parameter required to regenerate the synthetic corpus and to reproduce the analyses end-to-end. The supplementary material is organized as a single comprehensive table (Table S1) covering signal generation, signal-processing front end, fusion, features, classifier hyperparameters, validation procedure, statistical analysis, and the computational environment. References to the main-manuscript sections are given inline.

## Supplementary Table S1. Complete parameter listing for the representation-level wavelet–EMD fusion framework.

### S1.A — Synthetic pediatric EEG corpus (main manuscript §2.1, Table 3)

| **Parameter** | **Value or range** | **Comment** |
| --- | --- | --- |
| Sampling rate | 256 Hz | Fixed |
| Segment duration | 10 s | Non-overlapping epochs |
| Total segments | 2,000 | 1,000 epileptic + 1,000 normal |
| Class balance | 50% / 50% | Maintained across all CV folds |
| Delta band power | 20–40 µV² (default 30) | Sinusoidal components in 1–4 Hz |
| Theta band power | 15–30 µV² (default 22) | Sinusoidal components in 4–8 Hz |
| Alpha band power | 25–50 µV² (default 35) | Sinusoidal components in 8–13 Hz |
| Beta band power | 10–25 µV² (default 18) | Sinusoidal components in 13–30 Hz |
| Gamma band power | 5–15 µV² (default 8) | Sinusoidal components in 30–50 Hz |
| Phase model | Uniform on [0, 2π) per band per segment | Independent across bands |
| Spike amplitude *A*_spike_ | 50–150 µV (uniform) | Per spike, sampled independently |
| Spike decay constant α | 0.05–0.20 s^−2^ (uniform) | Controls 50–200 ms half-width |
| Spike frequency *f*_spike_ | 15–25 Hz (uniform) | Carrier frequency in Gabor kernel |
| Spike phase φ_k_ | Uniform on [0, 2π) | Independent per spike |
| Spike occurrence rate | 0.1–2.0 Hz (uniform per segment) | Events distributed via Poisson process |
| Spike-and-wave repetition | 2.5–4 Hz | Within spike-and-wave class only |
| Polyspike cluster size | 3–6 spikes at 4–6 Hz | Within polyspike class only |
| Slow-wave duration | 0.5–2 s | Within slow-wave class only |
| Slow-wave amplitude factor | 1.5–3× background | Relative to local RMS |
| Gaussian white noise | SNR 5–25 dB | Variance set per segment |
| Colored physiological noise | Pink-spectrum, 1/*f*, SNR 5–25 dB | Muscle-band proxy |
| Impulsive noise | Sparse spikes, 0.1–0.5 Hz, ±200–500 µV | Movement-artifact proxy |
| Mixed noise model | Convex combination of all three | Used for “mixed noise” robustness panel |
| Random seed (corpus generation) | 42 (master), per-segment seeds in repository | Documented in repository config file |
| Train / dev / eval split | 80% / 20% before CV; CV on 80% partition | Dev partition used for hyperparameter tuning only |

### S1.B — Wavelet preprocessing (main manuscript §2.2)

| **Parameter** | **Value** | **Comment** |
| --- | --- | --- |
| Mother wavelet | db4 (Daubechies-4) | Selected over bior3.5, coif3 (Table 1) |
| Decomposition levels | 6 | Yields scales 2 to 128 Hz at 256 Hz fs |
| Detail coefficients | *D*_1_ … *D*_6_ | Per-level retained |
| Approximation coefficient | *A*_6_ | Coarsest scale |
| Thresholding rule | Soft, per-level | τ_j_ = 0.05 · max\|*D*_j_\| |
| Reconstruction | Inverse DWT after thresholding | Mallat pyramid algorithm |
| Boundary handling | Symmetric extension | Equivalent to MATLAB wextend with mode sym |

### S1.C — Empirical mode decomposition (main manuscript §2.3)

| **Parameter** | **Value** | **Comment** |
| --- | --- | --- |
| Envelope interpolation | Cubic spline | Through local extrema |
| Boundary extension | Mirror extension at endpoints | Mitigates end effects in spline fit |
| IMF admissibility criterion 1 | \|#extrema − #zero crossings\| ≤ 1 | Standard EMD |
| IMF admissibility criterion 2 | Mean envelope = 0 (to numerical tolerance) | Tolerance 10^−6^ |
| Sifting termination (intra-IMF) | Standard deviation between successive sift iterations < 0.2 | Inner sifting loop |
| Sifting termination (across IMFs) | *R*_k_ = (*E*_k−1_ − *E*_k_) / *E*_k−1_ < 10^−3^ | Energy criterion (main text Eq. 4) |
| Maximum IMFs computed | 12 | Hard cap, rarely reached |
| Typical IMFs per segment | 6 to 8 | Observed empirically |
| IMFs retained for fusion | Top 4 by relative energy | Captures dominant oscillatory modes |
| Hilbert transform | Computed per retained IMF | Yields instantaneous frequency and amplitude |

### S1.D — Representation-level fusion (main manuscript §2.4)

| **Parameter** | **Value** | **Comment** |
| --- | --- | --- |
| Wavelet spectrum *S*_W_(*f*, *t*) | Squared magnitude of continuous-wavelet scalogram | Built on retained DWT scales |
| EMD spectrum *S*_E_(*f*, *t*) | Hilbert spectrum from retained IMFs | Energy density on (*f*, *t*) |
| Frequency grid | 64 linearly spaced bins in 1–64 Hz | Common to *S*_W_ and *S*_E_ |
| Time resampling | Linear interpolation to original 256 Hz grid | Aligns the two representations |
| Fusion weight α (wavelet) | 0.6 | Grid search on dev partition (0.3–0.7 step 0.05) |
| Fusion weight β (EMD) | 0.4 | β = 1 − α |
| Optimization objective | F1 score on dev partition | Section 2.4 |

### S1.E — Feature extraction and standardization (main manuscript §2.5, Table 2)

| **Parameter** | **Value** | **Comment** |
| --- | --- | --- |
| Number of features | 8 | See Table 2 for definitions |
| Spectral roll-off threshold | 85% cumulative power | Fixed |
| Spectral entropy normalization | *P̃*(*f*) = *P*(*f*) / Σ_f_ *P*(*f*) | Probability mass on the spectrum |
| Instantaneous bandwidth aggregation | Mean over time within segment | Single scalar per segment |
| Standardization | Zero mean, unit variance | Per-feature |
| Standardization scope | Per training fold only | No leakage across CV folds |
| Dimensionality reduction | None (PCA evaluated but not retained) | Section 2.5 |

### S1.F — Classifier hyperparameters (main manuscript §2.6, Table 7)

| **Component** | **Setting** | **Comment** |
| --- | --- | --- |
| Linear SVM — *C* | Grid {0.1, 1, 10, 100} | 3-fold inner CV |
| RBF SVM — *C* | Grid {0.1, 1, 10, 100} | 3-fold inner CV |
| RBF SVM — γ | Grid {10^−3^, 10^−2^, 10^−1^, 1} | 3-fold inner CV |
| RBF SVM probability calibration | Platt scaling | Logistic regression on scores |
| Random forest — trees | 200 |  |
| Random forest — max depth | 15 |  |
| Random forest — min samples per leaf | 2 |  |
| Random forest — feature subsampling | √*p* per split | *p* = 8 |
| Random forest — bagging | Enabled, 1.0× sample fraction with replacement |  |
| MLP — hidden widths | 64, 32 | Two hidden layers |
| MLP — activation | ReLU |  |
| MLP — dropout | 0.2 | Between hidden layers |
| MLP — optimizer | Adam |  |
| MLP — learning rate | 1 × 10^−3^ |  |
| MLP — batch size | 64 |  |
| MLP — early stopping | Patience 15 epochs on validation loss |  |
| Ensemble combination | Soft voting | Mean of calibrated posteriors |
| Ensemble component weights | (1/3, 1/3, 1/3) | Equal weights |
| EEGNet (pre-specified, §2.7.2) | F1 = 8, D = 2, F2 = 16; kernel 64; dropout 0.5 | Executed on synthetic corpus (§3.8, Table 10) |
| 1D-CNN (pre-specified, §2.7.2) | 3 blocks: filters 32/64/128, kernels 7/5/3, GAP+FC | Executed on synthetic corpus (§3.8, Table 10) |

### S1.G — Validation and statistics (main manuscript §2.8, §2.9)

| **Parameter** | **Value** | **Comment** |
| --- | --- | --- |
| Outer cross-validation | 10-fold stratified | Class balance preserved |
| Monte Carlo replications | 100 with independent seeds | For all reported mean ± SD |
| Inner cross-validation (hyperparameter) | 3-fold | On the 20% development partition only; selected values frozen for all evaluation |
| AUROC confidence interval | Bootstrap, 1,000 iterations | On pooled predictions |
| Method-comparison test | McNemar’s paired test | On per-segment predictions |
| Per-feature test | Mann–Whitney *U* test | Two-sided |
| Multiple-comparison correction (methods) | Bonferroni across the 6 pre-specified method pairs (proposed vs Fourier, wavelet, EMD and feature-level fusion; wavelet vs EMD; feature- vs representation-level fusion) | Adjusted α = 0.05 / 6 = 0.00833 |
| Multiple-comparison correction (features) | Bonferroni across 8 features | Adjusted α = 0.05 / 8 = 0.00625 |
| Effect size | Cliff’s δ | \|δ\| ≥ 0.474 interpreted as large |
| Master random seed | 42 | Per-fold and per-MC seeds derived |

### S1.H — Computational environment (main manuscript §2.10)

| **Component** | **Specification** |
| --- | --- |
| Numerical platform (proposed framework, baselines) | MATLAB R2023a with Statistics and Machine Learning Toolbox |
| Parallelization | MATLAB Parallel Computing Toolbox |
| Numerical platform (deep learning baselines, §2.7.2) | PyTorch 2.0 on Python 3.10 |
| CPU | Intel Xeon |
| System memory | 32 GB |
| GPU (for §2.7.2 deep learning) | NVIDIA RTX 4090, 24 GB VRAM |
| Operating system | Documented in repository |
| Random seed management | Master seed 42; per-experiment seeds in config files |
| Determinism | Single-threaded mt19937ar for CPU experiments |
| Software repository | GitHub, DOI mirrored on Zenodo at acceptance |

## Notes on use

Every parameter required to reproduce the synthetic corpus is in S1.A, with the noise models in S1.A and the wavelet/EMD pipeline in S1.B–S1.D.

Classifier hyperparameter values that were *searched* (rather than fixed) are listed with their search grids; the selected operating points are those reported in Table 1 of the main manuscript.

The EEGNet and 1D-CNN deep learning baselines are specified in §2.7.2 of the main manuscript and were executed on the synthetic corpus, with results reported in §3.8 (Table 10) and external results in §3.9 (Table 11); their full hyperparameter tables are reproduced here in S1.F so that the present supplementary file remains self-contained.

**Supplementary Table S2.** Permutation feature importance in the final soft-voting ensemble, reported as the mean decrease in AUROC (± SD) when each feature is independently permuted, on the synthetic corpus and on the CHB-MIT external evaluation. The ranking agrees with the univariate ordering of main-manuscript Table 8.

| **Rank** | **Feature** | **Synthetic ΔAUROC** | **CHB-MIT ΔAUROC** |
| --- | --- | --- | --- |
| 1 | Spectral entropy | 0.045 ± 0.009 | 0.036 ± 0.008 |
| 2 | Spectral centroid | 0.038 ± 0.008 | 0.031 ± 0.007 |
| 3 | Peak frequency | 0.032 ± 0.007 | 0.026 ± 0.006 |
| 4 | Spectral bandwidth | 0.026 ± 0.006 | 0.021 ± 0.005 |
| 5 | Zero-crossing rate | 0.022 ± 0.005 | 0.018 ± 0.005 |
| 6 | Spectral roll-off | 0.018 ± 0.005 | 0.015 ± 0.004 |
| 7 | Instantaneous bandwidth | 0.015 ± 0.004 | 0.012 ± 0.004 |
| 8 | Spectral flatness | 0.010 ± 0.003 | 0.008 ± 0.003 |

**Supplementary Table S3.** Robustness of the proposed framework to synthetic generator shift. Models were trained and tuned on the original synthetic distribution and evaluated on shifted distributions; values are mean ± SD across replications.

| **Test condition** | **Accuracy (%)** | **Sensitivity (%)** | **Specificity (%)** | **F1** | **AUROC** |
| --- | --- | --- | --- | --- | --- |
| Original synthetic distribution | 94.6 ± 0.6 | 93.7 ± 0.8 | 95.5 ± 0.5 | 0.943 ± 0.007 | 0.971 ± 0.005 |
| Shifted amplitude (spikes 35–120 µV) | 91.8 ± 0.9 | 89.6 ± 1.2 | 94.0 ± 0.8 | 0.914 ± 0.009 | 0.948 ± 0.008 |
| Shifted frequency (carrier 10–30 Hz) | 92.3 ± 0.8 | 90.8 ± 1.1 | 93.7 ± 0.9 | 0.920 ± 0.008 | 0.952 ± 0.007 |
| Increased artifact burden (×2 impulsive) | 89.7 ± 1.2 | 87.1 ± 1.5 | 92.3 ± 1.1 | 0.891 ± 0.012 | 0.927 ± 0.011 |
| Lower SNR (0–15 dB) | 88.9 ± 1.3 | 86.5 ± 1.6 | 91.2 ± 1.2 | 0.884 ± 0.013 | 0.918 ± 0.012 |
| Morphology imbalance | 90.6 ± 1.1 | 88.4 ± 1.4 | 92.8 ± 1.0 | 0.901 ± 0.011 | 0.935 ± 0.010 |
| Combined shift | 86.4 ± 1.6 | 83.8 ± 2.0 | 89.0 ± 1.5 | 0.856 ± 0.017 | 0.894 ± 0.015 |

**Supplementary Table S4.** Probability-calibration analysis. Brier score and expected calibration error (ECE) on the synthetic corpus and on the CHB-MIT external evaluation; lower values indicate better calibration.

| **Model** | **Synthetic Brier** | **Synthetic ECE** | **CHB-MIT Brier** | **CHB-MIT ECE** |
| --- | --- | --- | --- | --- |
| Fourier/Welch + ensemble | 0.186 | 0.092 | 0.226 | 0.128 |
| Wavelet alone + ensemble | 0.132 | 0.066 | 0.174 | 0.095 |
| EMD alone + ensemble | 0.151 | 0.074 | 0.191 | 0.104 |
| Feature-level fusion | 0.079 | 0.038 | 0.128 | 0.071 |
| EEGNet | 0.073 | 0.041 | 0.119 | 0.077 |
| 1D-CNN | 0.069 | 0.039 | 0.114 | 0.073 |
| Representation-level fusion (proposed) | 0.064 | 0.032 | 0.109 | 0.064 |

**Supplementary Table S5.** Per-subject leave-one-subject-out results on the CHB-MIT corpus for the proposed framework (14 subjects, 12,480 epochs), confirming that performance is not driven by any single subject.

| **Subject** | **Accuracy (%)** | **Sensitivity (%)** | **Specificity (%)** | **F1** | **AUROC** |
| --- | --- | --- | --- | --- | --- |
| S01 | 89.0 | 83.3 | 90.9 | 0.779 | 0.923 |
| S02 | 84.8 | 76.7 | 87.5 | 0.716 | 0.884 |
| S03 | 88.6 | 81.9 | 90.8 | 0.765 | 0.912 |
| S04 | 85.7 | 77.3 | 88.4 | 0.731 | 0.891 |
| S05 | 87.4 | 80.5 | 89.7 | 0.755 | 0.906 |
| S06 | 83.9 | 74.6 | 87.0 | 0.697 | 0.872 |
| S07 | 88.3 | 82.1 | 90.4 | 0.769 | 0.916 |
| S08 | 85.1 | 75.8 | 88.2 | 0.714 | 0.887 |
| S09 | 87.8 | 80.0 | 90.5 | 0.759 | 0.910 |
| S10 | 86.1 | 78.4 | 88.7 | 0.737 | 0.898 |
| S11 | 90.0 | 84.7 | 91.8 | 0.799 | 0.931 |
| S12 | 83.6 | 72.9 | 87.2 | 0.686 | 0.861 |
| S13 | 87.1 | 79.5 | 89.6 | 0.748 | 0.904 |
| S14 | 86.9 | 80.0 | 89.2 | 0.747 | 0.899 |
| Mean ± SD | 86.7 ± 2.0 | 79.1 ± 3.6 | 89.3 ± 1.5 | 0.747 ± 0.031 | 0.900 ± 0.020 |

**Supplementary Code 1.** Reproducibility code and configuration files for simulated EEG generation, wavelet–EMD fusion, model evaluation, and CHB-MIT external validation.

**Supplementary Figure S1.** Permutation feature importance in the final ensemble on the synthetic corpus and the CHB-MIT external evaluation, reported as the mean decrease in AUROC when each feature is independently permuted. Spectral entropy, spectral centroid and peak frequency are the strongest predictors in both settings.

**Supplementary Figure S2.** Robustness of representation-level fusion under synthetic generator shift. Bars show accuracy, sensitivity and specificity (left axis) and the line shows AUROC (right axis) for the original distribution and six shifted distributions; performance degrades gracefully and remains above the single-domain baselines in every condition.
